# The computationally guided design of selective targeters of multiple proteins (STaMPs) as a new opportunity for small molecule drug discovery

**DOI:** 10.3389/fphar.2025.1691119

**Published:** 2025-10-07

**Authors:** Thomas M. Kaiser

**Affiliations:** Avicenna Biosciences Inc., Durham, NC, United States

**Keywords:** synthetic biology, drug design, polypharmacology, computational drug design, machine learning, systems biology, medicinal chemistry, pleiotropism

## Abstract

Polypharmacology has long been an aspect of drug design for small molecules, and the multi-target pursuit has frequently behaved more akin to divine chance rather than controllable science. Targets unknown or once thought undesirable can often be revealed to be key points of intervention for the positive effects of a drug later in the development of a program or even after its approval. In this review, we look at historical examples of molecular pleiotropism and evaluate how new insights from computational systems biology and small molecule design can aid the rational design of Selective Targeters of Multiple Proteins (STaMPs).

## 1 Introduction

Medicinal chemistry arose out of the German dye industry at the turn of the 20th century, and the tuning of these chromophores into pharmacophores was guided at that time by phenotypic evaluation in animal models of disease (Manavi et al., 2024). The concept of DNA as the genetic code would be decades away (let alone the notion of proteins as targets) when the first synthetic drugs were discovered (Prescott, 2000). Paracetamol, still one of the most successful contemporary analgesics discovered in that early era, highlights how this early phenotypic process was able to produce extraordinary clinical utility without mechanistic insight into the mode of action (Graham and Scott, 2005). However, these early multi-target small molecules frequently had undesirable effects, and a movement emerged seeking to reduce the interactions of a ligand to a defined set of targets. The last two decades of the 20th century saw the emergence of “one-target-one disease” as the dominant philosophy of drug design with notable exceptions like antidepressant or antipsychotic drug design (Wermuth, 2004; Casas et al., 2019; Bartolomeis et al., 2022). A new appreciation of deliberate design of multi-target system modulation through a single molecule has appeared over the last 20 years of medicinal chemistry, though this multi-target approach is still not in the mainstream of drug design strategies (Proschak et al., 2019). The goals of this review are to define the properties of the modern, intentionally-designed pleiotropic drug, to highlight the potential therapeutic improvements of systems biology disruption, as well as highlight the new computational and data science tools like artificial intelligence/machine learning platforms which will make the design of such pleiotropic drugs facile.

## 2 Modern multi-target drugs

The shift from animal-based phenotypic screening to the “one-target, one-disease” approach to drug design has produced many effective drugs in the modern Pharmacopeia (examples of clinically transformative modalities include the BCR-Abl, Hepatitis C polymerase and TNFα inhibitors) (Martinelli et al., 2005; Sofia, 2016; Brekke and Sandlie, 2003). However, the focus on the design of highly specific agents which did not have the cross-reactivity of older medicines may have resulted in experimental therapeutics which were safe and yet not effective in clinical trials. As the single-target, hyperselective drugs designed in 80s, 90s and 00s moved into the clinic, the development success likelihood declined significantly (Smieta et al., 2016). Interestingly, there were far fewer failures in Phases I and II due to toxicological findings and far more Phase II failures due to lack of efficacy compared to earlier periods of development (Fogel, 2018). While there are some recent positive improvements in clinical trial success rates for Phase II and Phase III, it is still the case that experimental drugs have only a 10% success rate on average when entering clinical development which is much lower than 4–5 decades ago (Dowden and Munro, 2019). Economic factors related to the 2007 financial crisis could play a role in the decline in clinical success, although the sustained low success rate across therapeutic indications into the 2020s suggests that there are other factors causing the decline in translation success. One of these factors is that the deliberate design away from pleiotropism has reduced the ability of experimental drugs to meaningfully engage disease (Hopkins, 2007; Reddy and Zhang, 2013). As the clinical pharmacology and drug design communities bear down on diseases of unhealthy aging, neurodegeneration and inflammation, there has been renewed interest in systems biology and systems pharmacology. Designing agents which can engage multiple aspects of systems pathology in a deliberate and selective manner may offer a way to increase efficacy while not introducing limiting toxicological effects.

The pioneering work of Morphy and Rankovic published in 2004 first outlined the three approaches to polypharmacology (Morphy et al., 2004; Morphy and Rankovic, 2005). In this work, multitarget engagement could be achieved by (1) administering a drug cocktail, (2) a single drug comprised of multiple active pharmaceutical ingredients (e.g., Atripla^®^) or (3) a multiple ligand–a single small molecule drug that can modulate multiple targets concurrently. Since their original formulation, the idea of a multiple ligand has expanded beyond their original conception with emergence of targeting chimeras (TACs, e.g., PROTACs and AUTOTACs) and molecular glues: agents that indeed are multiple ligands but interact with their targets to generate synthetic biological complexes with the ability to induce degradation (vepdegestrant, a PROTAC) or stabilize macromolecular complexes (tacrolimus, a molecular glue) (Békés et al., 2022; Schreiber, 2024). Molecular glues and PROTACs are distinct from multitarget modulators in that multitarget modulators tend to be non-naturally occurring small molecules of a molecular weight <600 Da in a dissimilar chemical space from PROTACs and molecular glues (S et al., 2024; Apprato et al., 2024). Therefore, we wish to standardize a subtype of multiple ligand distinct from the PROTAC or molecular glue classes: the Selective Targeter of Multiple Proteins, or STaMP. The reason for this standardization is that molecular glues and PROTACs have distinct design criteria from STaMPs from both a chemistry perspective and a pharmacology perspective. Beyond the chemical space dissimilarities between PROTACs, molecular glues and STaMPs, a PROTAC or molecular glue may functionally target only a single point of intervention in a way that does not engage multiple points of a pathological system. This distinction in mechanism will impact the computational methods considered for STaMP design versus PROTAC or molecular glue design.

In order to highlight new techniques for the design of STaMPs, this manuscript defines a STaMP according to the framework outlined in Table 1. The goal of the range of Targets and Off-Targets is to include the atypical antipsychotics within the STaMP definition; (University of North Carolina at Chapel Hill and the United States National Institute of Mental Health) although, the number of targets modulated by the atypicals may be an outlier as this review anticipates that modern antidegeneration STaMPs will have between 2 and 7 low nanomolar target/off-target interactions in total. The proposed profile in Table 1 is for a compound with typical chemical properties for small molecule medicinal chemistry with respect to molecular weight, lipophilicity and other chemical constraints (Di and Kerns, 2016). Additionally, the number of targets/off-targets that are maximally permissible for a STaMP drug discovery campaign will depend on the targets’ physiology and any associated synergistic toxicology (Kabir and Muth, 2022; Rao et al., 2019). While there will likely be opportunities for STaMP design against single cellular types (e.g., survival pathway disruption in oncology) (Lazarte et al., 2025), this review proposes that a STaMP will find maximum therapeutic impact by disrupting pathological systems across cell lineages involved in disease (e.g., neuroinflammation, glial dysfunction and neural pathology in some types of neurodegeneration) (Castro-Gomez and Heneka, 2024; Stevenson et al., 2020).

**TABLE 1:** The proposed profile of a selective targeter of multiple proteins (STaMP).

Property	Range	Commentary
Molecular weight	<600	This range will be highly conditional on target organ compartment and chemical space
Number of targets	2–10	Potency for each should be at least <50 nM
Number of off-targets	<5	Off-Target is defined as a target with an IC_50_ or EC_50_ <500 nM
Number of cellular types which are being targeted (excepting oncology)	≥1	Multiple cell types involved in a disease process should be addressed with a single compound

## 3 Target combination identification for STaMPs

With this definition of a STaMP established, we turn our attention to highlighting key challenges in design as well as providing a survey of computational techniques which can aid in the rational design of these selective, multi-target ligands. Multifactor processes involved in the pathogenesis of metabolic diseases, neurodegeneration and inflammatory disorders are increasingly being understood in terms of systems biology and the interplay between several molecular partners (Proschak et al., 2019; Lillich et al., 2021; Artasensi et al., 2020; Ramsay et al.; Ma et al., 2020; Cavalli et al., 2008; Jana et al., 2022; Diaz-Beltran et al., 2013). The first key aspect to the development of a STaMP is the identification of biological target combinations which offer synergistic disease antagonism when those targets are modulated in the correct combination. This synergistic modulation may occur in a variety of cellular types in a tissue or an organ, and key considerations here will be considered in the review of technologies which can aid the design of STaMPs. The focus of this section will be on the developments for target selection that have been achieved across the systems biology, multi-omics and machine learning spaces, and how these new techniques offer promise for the design of STaMPs. Additionally, there are informatics approaches which can make use of clinical pharmacology databases or screen approved drug combinations in a high throughput format, however these methods have been extensively reviewed elsewhere (Proschak et al., 2019).

One of the emerging areas for the identification of targets for pleiotropic agents is the development of integrative -omics techniques (Noor et al., 2023). Transcriptomics, proteomics and metabolomics evaluate large scale biological experiments for changes in transcription, protein interaction and metabolism (Hasin et al., 2017). Although, the ability to identify key nodes of disease in patient samples and decipher the complexities of pathology in biological systems across biological layers (proteins, mRNA, etc.) remains a fundamental challenge for the -omics approach. That being said, significant progress in the integration of -omics data sets to provide novel biological insight to disease processes has been accomplished through the use of network analysis and machine learning (Borrego-Yaniz et al., 2024; Sanches et al., 2024). There are several excellent reviews for designing multi-omic studies, and these reviews offer mitigating strategies for dealing with potential pitfalls due to data heterogeneity, noise versus biological variation and poorly formulated questions for a given experimental technique (Borrego-Yaniz et al., 2024; Paananen and Fortino, 2019). Examples of correlation-based approaches for biological network analysis include Cytoscape (gene-metabolite method) (Cline et al., 2007) and Similarity Network Fusion methods (various -omics data networks created independently and then iteratively merged across biological layers) (Wang et al., 2014; Mu Yang et al., 2023). The work of Felsky and colleagues has demonstrated multi-omic integration by Similarity Network Fusion as a capable technique for analyzing human frontal cortex samples for -omics molecular subtypes associated with cognitive decline and neuropathology (Mu Yang et al., 2023). Additionally, Martin et al. explored the utility integrative-omics methods when applied to immune-mediated inflammatory diseases and reported that these approaches offer a comprehensive platform for understanding how multiple biological targets are involved in complex, multifactor disease processes across changes in lipids, proteins, transcription events and epigenetics (Borrego-Yaniz et al., 2024).

While network analysis methods can serve as an isolated method for multi-omics, the advent of machine learning applications in bioinformatics can aid in causal linkage discovery and/or integration between-omics and clinical parameters like demographics, presentation and outcome (Casas et al., 2019; Stafford et al., 2020; Jiang et al., 2017). This was the case with systemic autoimmune disease (SAD) patients where an unsupervised clustering method was used to integrate whole blood transcriptome and methylome data of SAD patients for seven types of systemic inflammatory disease (e.g., people with systemic lupus, rheumatoid arthritis, Sjögrens’s and systemic sclerosis) and healthy volunteers (Barturen et al., 2021). This revealed three distinct molecular clusters termed inflammatory, lymphoid and interferon which were stable across the seven clinical diagnoses. Not only were there discrete target sets enriched in each distinct cluster, but the three clusters were stable over time suggesting these clusters to be related to the fundamental disease biology for systemic inflammation. This categorization into potential multi-target phenotypes is therefore not only important for drug design from a preclinical perspective for STaMP design, but it is also important from a patient recruitment perspective for eventual clinical trials. These unsupervised clustering methods that can be used on both patient and animal samples can ensure that the STaMP target pleiotropic profile is linked with a disease model that shares the same molecular fingerprint as the eventual patient population cluster, given patient cluster subtypes will respond to therapies at differing rates (Khamashta et al., 2016).

Complementary to the multi-omics approaches reviewed above, there is also a large body of work evaluating protein-protein interactions (PPIs) as a key area to identify new targets involved in the systems biology of disease. The work of target identification through genome-wide association studies is important and impactful for finding drugs (Trajanoska et al., 2023; Nelson et al., 2015; Floris et al., 2018; Namba et al., 2022); however, work has demonstrated that PPI networks can reveal potentially druggable protein targets that, while not directly genetically associated themselves, are key points of interaction for targets which are genetically associated and yet less easily druggable (Fang et al., 2019). Fang et al. demonstrated that integration of functional genomic data and PPI networks led to a discovery of a set of targets that were key points of interaction with genetically linked proteins in a GWAS, and these identified nodes could be successfully modulated in cellular models of inflammatory disease (e.g., ICAM1 and its interaction with RhoA in ankylosing spondylitis, systemic lupus erythematosus and juvenile idiopathic arthritis, among others) (Fang et al., 2019). Interesting informatics methods for validating targets identified through genome-wide association studies and/or protein-protein interaction networks are being developed alongside the target identification methods discussed. A high throughput method for combining genetic evidence for potential targets and CRISPER/Cas9 modulation of these targets was developed by Yu et al. which allowed for the evaluation of candidate target according to their biologically importance for hepatic stellate cell activation in the presence of TGF-β stimulation (Yu et al., 2022). This work along with additional systems biology work could serve as an excellent starting point for exploring synergistic targets of interest for STaMP design to disrupt the multifactor fibrosis cascade driving liver fibrosis in advanced liver disease (Bashir et al., 2022).

As important as it is to select the targets one ought to modulate, the targets that a compound should avoid interacting with at physiologically relevant concentrations can be a difficult and yet equally important design consideration to address. While clear off-targets have emerged like hERG, it is often the case that desirable targets related to the pathogenesis of a disease of interest have dose-limiting on-target toxicological effects which preclude clinical development against that target (Lin et al., 2019; Bendels et al., 2019). Machine learning approaches are being tailored for predicting the toxicology of a target of interest, and Gao et al. have explored a deep learning approach using Genetic Profile-Activity Relationships (GPAR) to predict toxic phenotypes given some input chemical structure with reported examples of serotonin transporter inhibition, Na^+^/K^+^ ATPase inhibition and NF-κB inhibition being evaluated as use cases (Gao et al., 2021). While the method of Gao et al. requires correlating the changes in gene expression data when a cellular is exposed to a compound’s structure, there are emerging techniques employing the same gene expression data that evaluate targets for possible toxicology independently of chemical structure (Masarone et al., 2025). The use of networks across multi-omics information can also be useful for building methods like ComptoxAI which attempts to link chemical modulation with pathways and systems that account for an observed toxicologic effect (Romano et al., 2022). Although Comptox also requires input structures like the method of Gao et al., this review proposes that a candidate target of interest regularly implicated in similar toxicology across a variety of pharmacophores by ComptoxAI would allow the target to be deprioritized for STaMP design. Additionally, there have been advances in employing transcriptomics techniques which can evaluate a toxicogenomics phenotype consisting of more than 1,300 genes and involving 250 million datapoints to construct a predictive toxicogenomic space (PTGS) which could be employed to predict liver toxicity for STaMPs which are being designed at the discovery stage (Kohonen et al., 2017). While this work is an impressive initial inroad to anticipating liver toxicity, complex, organ-specific toxic events like drug-induced liver injury can be difficult to reduce to a single target (Yuan and Kaplowitz, 2013; Andrade et al., 2019). Unfortunately, a reductionistic method that can predict synergistic target combinations causing organ toxicity is not yet available. Several methods exist for predicting toxicology for chemical structures across a variety of organs without respect to the targets of said strucutres (Cavasotto and Scardino, 2022), but teasing out potential toxic combinations of drug targets remains a key area of interest for the development of computational methods to aid in the design of STaMPs.

## 4 Techniques for the design of STaMP ligands across biological targets

Ample chemical starting points exist for the design of a drug be the drug profile selective or pleiotropic. This is such the case that the true size of the space that contains all drugs be they natural product or synthetic is unintelligible (Lachance et al., 2012; Reymond, 2015). Given the synthetic inefficiency that currently limits natural product modification (despite the utility for new medicine discovery) (Truax and Romo, 2020), we accordingly constrain ourselves to synthetic molecules as opportunities for fine-tuned and rational STaMP design and will therefore limit this section to the review of methods which can be impactful in the synthetic small molecule chemical space.

Physics-based methods have made major improvements in accuracy over older methods used to predict potency against a biological target (e.g., GLIDE) (Jorgensen and Thomas, 2008; Kirkpatrick, 2004; Abel et al., 2017). These advances in methods like thermodynamic integration (TI) and free energy perturbation (FEP) have been driven by an increase in computing power serving both the refinement in methodology as well as improving the compound throughput markedly (Harder et al., 2016; Damm et al., 2025; Giese and York, 2018; Lee et al., 2017). Importantly for the design of STaMPs, these improvements allow physics-based methods to serve both a “dial-in” approach where additional target affinities are designed into a given scaffold and a “dial-out” approach where undesired target affinities are removed through the use of computational techniques. While limited examples exist of FEP being used to rationally design STaMPs, the work of Patel et al. highlights the utility of FEP in balancing affinity against two desired targets: human monophosphate kinase and hepatitis C viral RNA-dependent RNA polymerase (Patel et al., 2022). Antiviral nucleoside prodrugs must be converted to active triphosphates by a host nucleoside monophosphate kinase in order to be active against the viral polymerase, and the work of Patel et al. demonstrated that FEP could be employed to rationally design the multi-target constraints of a drug design program.

Machine learning has also been applied extensively for designing potency in concert with balancing other drug-like properties (Kosu et al., 2020; Antontsev et al., 2021; Kaiser et al., 2020; Han et al., 2023; Fowles et al., 2025). The ability to navigate large datasets and extract chemically meaningful information for the drug design problem of interest is a strength of machine learning, as long as the experimenter is careful with question phrasing. An example of machine learning in designing STaMP-like compounds was conducted by Bajorath et al. where they employed random forests to design dual monoamine oxidase B-acetylcholinesterase inhibitors (Feldmann et al., 2021). Here, the team focused on determining coherent substructures shared by ligands across both targets, and it was found that explainable ML methods were capable of building a bridge between chemical intuition and rational drug design. Although machine learning requires information on which to train, recent work has explored the utility of physics-based methods for augmenting machine learning datasets to facilitate machine learning algorithm construction (Burger et al., 2024; Ramaswamy et al., 2025; Thompson et al., 2022; Lonsdale et al., 2025). Such a hybrid approach will facilitate either “dial-in” or “dial-out” approaches for the design of STaMPs where there exists either a novel desirable target, or new insights reveal a target to be undesirable.

## 5 Conclusion

Given the stupefying progress achieved in the just the past decade with regard to informatics, -omics and computation, there is a great opportunity for the rational design of modern multi-target small molecules defined here as STaMPs. New insights in systems biology, mulit-omics, physics-based methods and machine learning can be employed to identify not only synergistic target combinations but also molecular targets with potentially toxic effects. Additionally, advances in computational methods can make it more efficient to optimize a chemical series for an experimentally determined target profile. While serendipity will always play a role in the discovery of new medicines, the technologies reviewed herein point to a new kind of medicinal chemistry: the rational design of STaMPs through advanced computation.
